# Implementation research is crucial to countries’ efforts to strengthen learning health systems

**DOI:** 10.1093/heapol/czaa119

**Published:** 2020-11-06

**Authors:** Abdul Ghaffar, Soumya Swaminathan, Kabir Sheikh, Ahmed Al-Mandhari, Manoj Jhalani, Boureima Sambo, Zsuszanna Jakab

**Affiliations:** 1 Alliance for Health Policy and Systems Research, World Health Organization, 20 Avenue Appia, 1217 Geneva, Switzerland; 2 World Health Organization, Geneva, Switzerland; 3 Regional Office for the Eastern Mediterranean, World Health Organization, Cairo, Egypt; 4 Regional Office for South East Asia, World Health Organization, New Delhi, India; 5 Country Office for Ethiopia, World Health Organization, Addis Ababa, Ethiopia

## Implementation research shines a light into the ‘black box’

Implementation barriers can be significant impediments to the performance of health systems in low- and middle-income countries (LMICs) and the achievement of broader goals such as Universal Health Coverage (UHC) (Santos *et al.*, 2017). These barriers often reflect local contextual issues and have a direct impact on the performance of programmes and policies. Sometimes they are traceable to underlying systemic issues—for instance, prevailing incentive structures for health workers may be encouraging them to over-report or under-report data, or past adverse experiences may have caused communities to lose trust in government health facilities. Such barriers are common and often possible to resolve—but only once they have been identified. When well-designed policies and programmes fail to achieve intended outcomes, implementation research (IR) shines a light into the implementation ‘black box’ and helps identify and explain the reasons behind implementation failures and find and test strategies to overcome them. In this way, IR converts local, context-specific knowledge that is often hidden and tacit into systematic learning that can help health systems improve the delivery of policies and programmes (Alonge *et al.*, 2019).

The task of generating and using relevant local knowledge cannot take place without an integral role for health system decision-makers (policymakers, programme managers and implementers) in the research process (Tricco *et al.*, 2018)—including defining the research agenda, co-producing the research with professional researchers, deliberating on the applicability of research findings and disseminating and utilizing the findings. Integrating health systems decision-makers into the research processes (also known as ‘embedding’) empowers them by equipping them with the skills and understanding of research processes, enhances their ownership of the research and, most importantly, increases the utilization of research findings for policy and programme improvements (Langlois *et al.*, 2019).

## A process for decision-maker-led IR

This approach of engaging health systems leadership in IR has been and remains central to WHO’s efforts in the area and is reflected in the design and practice of several key initiatives. These include the Implementation Research Platform which supported several rounds of LMIC led IR commencing in 2010, the Implementation and Delivery Science (IRDS) Collaboration, which brings together a range of multilateral and bilateral agencies and research institutions to promote IR, and a series of decision-maker-led IR initiatives spanning over 40 countries, supported by the Alliance for Health Policy and Systems Research in collaboration with diverse partner organizations.

**Figure 1:**
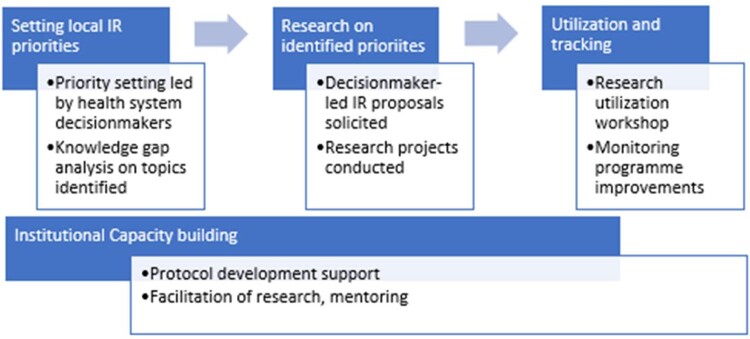
Decision-maker-led IR model advances local ownership and systems learning.

Through the experiences of these diverse initiatives over the past decade, an established process to engage and ensure local health systems leadership of the research has emerged (see Figure 1). This process typically starts with setting research priorities, in which in-country stakeholders, led by health system decision-makers from national and subnational levels, identify and prioritize key implementation barriers for the policy or programme in question, and draw on these challenges to develop priority research questions. The next step is to pair teams of decision-makers and in-country researchers to conduct collaborative research on the priority questions identified. The third step is to ensure that research findings are disseminated and applied to achieve programmatic improvements. Underpinning this process at every step is the crucial element of capacity building, through targeted workshops and mentorship support. The application of this model has frequently yielded timely knowledge to inform programme and policy action, and also contributed to learning in other ways.

For instance, a recent initiative of country-led IR for UHC engaged four Ministries of Health (Nepal, India, Ethiopia and Pakistan) on dedicated IR programmes to inform major national health programmes and policies focused on advancing UHC. Research findings from the initiative helped the Indian National Health Authority to (1) improve programme awareness among beneficiaries of the PMJAY national health insurance scheme, (2) strengthen and streamline processes related to beneficiary hospitalization and (3) strengthen structures and functions of institutions responsible for strategic purchasing across Indian states. The research in Pakistan has previously helped address bottlenecks in vaccine equity and access, and a new programme of IR will now support the delivery of the *Sehat Sahulat* social health protection scheme. In Nepal, the initiative has built the capacity of local organizations in research management as well as in the conduct and utilization of IR for UHC, based on research priorities identified by decision-makers. In Ethiopia, the initiative is focused on strengthening immunization services and the national scheme for a compassionate respectful and caring health workforce, for which research priorities were established by stakeholders from the Ministry of Health. Research teams combined their fieldwork with supporting community learning on COVID-19 prevention and testing.

## IR for the learning health system

Health systems learn in different ways—including learning through information, through deliberation and through action (AHPSR, 2021). IR has the potential to contribute in each of these learning dimensions—it is a crucial source of information about programme and policy actions, and involves stakeholder deliberations on research priorities, research processes and the utilization of research findings. Alongside, strengthening the utilization of routine health information, embedding IR into health systems is a crucial foundation stone for developing a learning health system (Sheikh *et al.*, 2020).

Never has the need for countries to develop learning health systems been more evident than in the case of the ongoing COVID-19 pandemic. Unfortunately, many LMIC health systems have not yet developed the capacities for integrating IR and collecting and using routine data to generate the intelligence needed for them to continuously respond, adapt and improve the current response (Yapa and Bärnighausen, 2018). Learning is currently low in the list of priorities for country health planners, and investments in health systems research and evaluation tend to be a very small proportion of overall investments in health programmes and systems. The pandemic reveals an emergent necessity to develop learning capacities within health systems. It is important that countries consider integrating IR into their major health programmes, ideally supported through domestic funds to ensure self-reliance and sustainability. IR has a crucial role in helping overcome barriers to programme and policy success, and as importantly, in engendering learning health systems with the inbuilt ability to generate the knowledge they need to constantly improve and perform.

## Disclaimer

The authors are staff members of the World Health Organization and are themselves alone responsible for the views expressed in the article, which do not necessarily represent the views, decisions or policies of the World Health Organization.

Abraham Assan provided valuable assistance in the preparation of this manuscript.

*Conflict of interest statement.* None declared.

*Ethical approval.* No ethical approval was required for this study.
